# Supporting the mental wellbeing of aged care workers: A systematic review of factors and interventions

**DOI:** 10.3934/publichealth.2025032

**Published:** 2025-06-17

**Authors:** Louise A Ellis, Tanja Schroeder, Maree Saba, Kate Churruca, Janet C Long, Annie Haver, Kristin Akerjordet, Kristiana Ludlow, Inger Johanne Bergerød, Sini Nevantaus, Jan-Willem Weenink, Zoe Gonzales, Samantha Spanos, Hilda Bø Lyng, Cecilie Haraldseid-Driftland, Daniel Adrian Lungu, Malin Knutsen Glette, Mari Lahti, Florin Tibu, Andreas Chatzittofis, Juana Maria Delgado-Saborit, Eila Kankaanpää, Viviana Wuthrich, Robyn Clay-Williams, Jeffrey Braithwaite, Siri Wiig

**Affiliations:** 1 Centre for Healthcare Resilience and Implementation Science, Australian Institute of Health Innovation, Macquarie University, Australia; 2 Lifespan Health and Wellbeing Research Centre, School of Psychological Sciences, Macquarie University, Sydney; 3 NHS-Department of Leadership and Service Innovation, Faculty of Social Sciences, University of Stavanger, Norway; 4 SHARE ‑ Centre for Resilience in Healthcare, Faculty of Health Sciences, University of Stavanger, Norway; 5 Centre for Health Services Research, the University of Queensland, Australia; 6 Faculty of Health and Well-Being, Turku University of Applied Sciences, Finland; 7 Erasmus School of Health Policy & Management, Erasmus University Rotterdam, Netherlands; 8 Faculty of Health, Western Norway University of Applied Sciences, Norway; 9 Faculty of Medicine and Biological Sciences, Ştefan cel Mare University of Suceava, Romania; 10 Medical School, University of Cyprus, Nicosia, Cyprus; 11 Department of Medicine, Faculty of Health Sciences, Universitat Jaume I, Spain; 12 Department of Health and Social Management, University of Eastern Finland, Finland

**Keywords:** mental health, mental wellbeing, staff burnout, emotional exhaustion, residential aged care, nursing homes, elderly care, aged care workers

## Abstract

**Background:**

The aged care sector faces significant challenges due to rising demand from aging populations and chronic diseases, in addition to workforce shortages, contributing to staff stress, burnout, and poor mental wellbeing. In this review, we synthesized quantitative studies on factors and interventions to improve the mental wellbeing of the aged care workforce across indicators and system levels.

**Methods:**

Five academic databases (Medline, Embase, Scopus, PsycInfo, and CINAHL) were searched from January 2014 to May 2024, using keywords related to aged care, care workers, and mental wellbeing. Quantitative studies examining factors or outcomes of interventions related to staff wellbeing were included. Identified factors were classified as micro-, meso-, and macro-level using a combined inductive and deductive approach.

**Results:**

Eighty-nine studies were included: 64 (72%) identified significant factors, and 25 (28%) evaluated interventions aiming to improve workers' mental wellbeing. Almost half concentrated on nursing staff (*n* = 38, 43%), with others addressing direct care workers (*n* = 25, 28%) or aged care workers more broadly (*n* = 19, 21%). From the synthesis, a multi-level model of factors affecting aged care worker wellbeing was developed, comprising 11 themes and 39 sub-themes; 3 micro-level themes (1. personal factors, 2. work engagement, and 3. skills and abilities), 6 meso-level themes (4. job demands, 5. professional relations, 6. job control, 7. leadership, 8. professional development, and 9. workplace resources), and 2 macro-level themes (10. policy and regulation and 11. structure and governance). Among the 25 intervention studies, most entailed micro-level changes (*n* = 24, 96%), including relaxation, emotion regulation, and behavioral management education training.

**Conclusions:**

Understanding system-level factors is a key to designing comprehensive improvement approaches. Our new model can help guide organizations in developing targeted strategies to promote mental wellbeing and strengthen care delivery. While individual-focused interventions have shown benefits, organizational and broader system-level strategies to improve mental wellbeing are pivotal for achieving sustainable change.

## 1. Introduction

The aged care sector faces significant challenges due to rising service demands driven by aging populations, chronic diseases, workforce shortages, and the ongoing impacts of the COVID-19 pandemic [1], all of which have exacerbated staff stress and burnout [2]. During the pandemic, the workload of aged care workers increased substantially; they experienced intensified emotional demands while feeling undervalued by the wider community [3]. With no respite from the intense stress experienced during the pandemic, aged care workers were required to continue ‘business as usual’ while service demands increased and staff shortages added pressure to the working environment [4]. Researchers have identified that workforce shortages in the aged care sector can be attributed to longstanding issues including heavy workloads, psychological and emotional stress, insufficient time to complete care activities, working in social isolation, poor organizational support, and lack of education and training [2],[5],[6]. In this challenging environment, aged care workers have been found to have poorer mental wellbeing, higher levels of burnout, and lower job satisfaction [2].

The World Health Organization (WHO) defines mental health as “a state of mental wellbeing that enables people to cope with the stresses of life, realize their abilities, learn and work well, and contribute to their community” [7]. Mental health can be viewed as a complex continuum from optimal mental wellbeing to severe psychological distress [8]. Reports indicate that during the COVID-19 pandemic, healthcare workers across the sector experienced symptoms such as traumatic stress, depression, anxiety, and emotional exhaustion, especially among those in direct contact with infected patients and those facing heavy workloads [9],[10]. The challenges encountered during the pandemic have resulted in long-term mental health consequences, contributing to increased job turnover and further strain on an already vulnerable workforce [11].

In recent years, researchers have synthesized the factors associated with poor mental wellbeing among aged care workers. These reviews have primarily concentrated on one or two specific indicators of mental wellbeing, most commonly burnout [2],[12]–[15]. Burnout is a psychological syndrome arising as a response to chronic, prolonged job-related stress, characterized by emotional exhaustion, depersonalization, and a low feeling of accomplishment [16]. A previous review of 14 studies in nursing homes and residential aged care facilities (RACFs) revealed burnout as a significant problem amongst aged care staff globally, with implications for the wellbeing of patients, providers, and staff. Identified factors associated with burnout included occupational aspects, such as high workloads and time pressures, as well as the types of coping mechanisms staff employ [14]. In another review of 21 studies [15], several factors associated with burnout were identified that encompassed individual factors, such as at-risk personality types, and organizational-related factors, such as lack of training, conflicts in the workplace, and high workload [15]. The implications of burnout among aged care workers are far-reaching; research shows that burnout contributes to staff depression and suicide, has strong links with staff work effort and retention, and adversely affects the quality of patient care and safety [17].

With growing recognition of the challenges facing the aged care workforce, there has been an increased focus on identifying strategies and interventions to improve staff mental health and wellbeing [18],[19]. Responding to these challenges requires a multi-level approach, focusing on individual (micro), organizational (meso), and broader system-level (macro) strategies [17]. However, much research on wellbeing interventions for aged care workers has concentrated on the individual-level (e.g., strategies to improve workers' coping skills), with limited evidence for interventions focused on the organizational or broader system-levels [19].

To our knowledge, no systematic review has synthesized contributory factors and interventions for aged care workers across indicators on the continuum of mental wellbeing and across system levels (micro, meso, and macro). Much of the research on aged care workers has focused on nurses [20], with limited research including personal care workers [21],[22], who make up 70% of the aged care workforce [23]. We sought to include studies of all members of the aged care workforce, including personal care workers who play a vital role in delivering frontline care to older adults.

### 1.1. This study

Our aims of the review were to:

1. Examine the empirical literature to identify micro-, meso-, and macro-level factors impacting the mental wellbeing of the aged care workforce; and
2. Examine the effectiveness of workplace interventions aiming to improve mental wellbeing of the aged care workforce. Owing to the heterogeneity of included wellbeing indicators, the purpose here was not to conduct a meta-analysis, but to identify and describe the intervention study results.

## 2. Methods

We followed the Preferred Reporting Items for Systematic Reviews and Meta-Analyses (PRISMA) guidelines (see Supplementary File 1) [24]. The review was pre-registered on PROSPERO (protocol number: CRD42024552679).

### 2.1. Search strategy

Five academic databases (Medline, Embase, Scopus, PsycInfo, and CINAHL) were searched on May 17, 2024. The search strategy was restricted to English-language articles published in the last 10 years (from January 1, 2014), and used terms pertaining to elderly care (e.g., aged care, elderly care, residential aged care, and nursing home); aged care workers (e.g., health personnel or nurses); and mental wellbeing (e.g., mental health, burnout or distress). The search strategy was developed in consultation with an academic research librarian and adapted for each database as necessary (see Supplementary File 2 for the complete search strategy).

### 2.2. Inclusion and exclusion criteria

Articles were included if they were: (a) Peer-reviewed journal articles with full-text available in English; (b) quantitative empirical studies; (c) conducted in an aged care setting; this included care homes for older people (aged 65 years or over), RACFs (also termed residential care homes, aged care homes, residential long-term care, or nursing homes), and home care (i.e., care services provided in the homes of older adults); and (d) reported on contributory factor/s or intervention outcomes relating to staff mental wellbeing (excluding informal/unpaid carers). Study protocols, review articles, journal commentaries and editorials, conference papers, doctoral dissertations, and book chapters were excluded. Studies entailing multiple care settings (e.g., nursing homes and hospitals) were also excluded.

### 2.3. Eligibility screening

Reference details (including abstracts) were downloaded into the reference management software EndNote (version 20.6) [25] and then exported to Rayyan QCRI [26] for title and abstract screening. A random sample of 10% of titles/abstracts was blind screened by twelve reviewers (LAE, KA, TS, SN, JL, JWW, KC, AH, KL, IJB, MS, and ZG) to ensure consistent application of inclusion and exclusion criteria. Discrepancies were discussed in a team meeting until a consensus was reached. A further 30 articles were blind screened by the review team, with Fleiss Kappa calculated as excellent at 85%. Six pairs of reviewers (LAE/KA, TS/SN, JL/JWW, KC/AH, KL/IJB, and MS/ZG) then independently screened the remaining titles/abstracts to determine their inclusion against the criteria. Included articles from title/abstract screening underwent a full-text review independently by ten reviewers. As a final step, TS and ZG checked all articles excluded at the full-text stage to confirm exclusion decisions and resolved conflicts in consultation with the wider review team. Regular meetings were held among the review team to ensure the consistency of article inclusion.

### 2.4. Data extraction

A customized data extraction workbook was developed in Microsoft Excel and independently piloted by 12 reviewers with a subset of papers (*n* = 5). Issues in the consistency of data entry and usability of the workbook were then discussed, and modifications were made accordingly. The remaining articles were distributed among the 12 reviewers for data extraction. Key information extracted included: Article characteristics (authors, date of publication, country of corresponding author, and journal name); participants (e.g., nursing staff, personal care workers, and all direct care workers); study setting (e.g., aged care facility/nursing home, and home care); study design (e.g., cross-sectional, longitudinal/cohort, and intervention); study methods (e.g., survey and mixed-methods); mental health outcomes (e.g., burnout, stress/distress, emotional/mental wellbeing, depression, and anxiety); and factors of mental wellbeing (i.e., micro, meso, and macro). Due to the large volume of data, only significant factors were extracted where the outcome variable was related to a measure of mental wellbeing (i.e., significant independent variables from the included studies).

For intervention studies, information regarding the following was also extracted: Intervention type (micro: Individual-directed; meso: Organization-directed; and macro: Broader system-directed); intervention study design (randomized controlled trial, non-randomized controlled trial, and longitudinal study without control); intervention duration, and last follow-up outcome measurement (short-term: End of intervention or less than one month after intervention; medium term: One month to eleven months after intervention; and long term: 12 months or longer). The data extracted for intervention studies were in accordance with the classification criteria for intervention studies in previous research [15],[27].

### 2.5. Quality assessment

The methodological quality of the studies was evaluated using the Joanna Briggs Institute (JBI) critical appraisal tools, including the JBI checklists for Analytical Cross-Sectional Studies, Cohort Studies, Quasi-Experimental Studies, and Randomized Controlled Trials (RCTs) [28]–[31]. The JBI tools were selected according to the design of each study assessed, with all items in the corresponding checklist being utilized. The tools were piloted by two independent reviewers (TS, ZG) with a subset of studies (*n* = 9); discrepancies in appraisals were managed through discussion between the reviewers to reach agreement. The remaining articles (*n* = 80) were critically assessed independently by two reviewers (TS, ZG), with 5 randomly selected studies assessed by a third reviewer (LAE). Score classifications were made using predetermined thresholds [32]: High quality for studies with over 70% “yes” scores; medium quality for studies with “yes” scores between 50% and 70%; and low quality for studies with “yes” scores below 50%. All studies, irrespective of quality, were included.

### 2.6. Data synthesis and analysis

Identified significant factors of mental health were initially classified into themes relating to micro- (individual), meso- (organization), and macro- (broader system) level factors. Similar factors were then grouped using an inductive approach by 3 authors (LAE, TS, and MS). The final categorizations of factors were developed through a combined inductive/deductive approach, drawing on the National Academy of Medicine's (NAM) Conceptual Model for Clinician Wellbeing and Resilience [33]. In 2017, the NAM established an Action Collaborative of leaders and representatives from more than 200 health professional organizations that developed the model designed to illustrate and guide the understanding of the factors that contribute to clinician wellbeing. While several conceptual frameworks of care worker mental wellbeing currently exist, the NAM model is among the most comprehensive, encompassing both individual factors (role, personal factors, skills, and abilities) and system factors (e.g., organizational factors and learning/practice environment) influencing staff mental wellbeing [34]. Additionally, the NAM model is inclusive of a broad range of professions and settings [34]. A collaborative whole-team workshop was held to review the preliminary results, culminating in the development of a coding framework with themes separated at three levels: Micro, meso, and macro. This coding framework included all identified themes and their corresponding sub-themes (see Supplementary File 3). The results were then recoded according to this coding framework and then narratively synthesized.

## 3. Results

### 3.1. Overview

**Figure 1.**
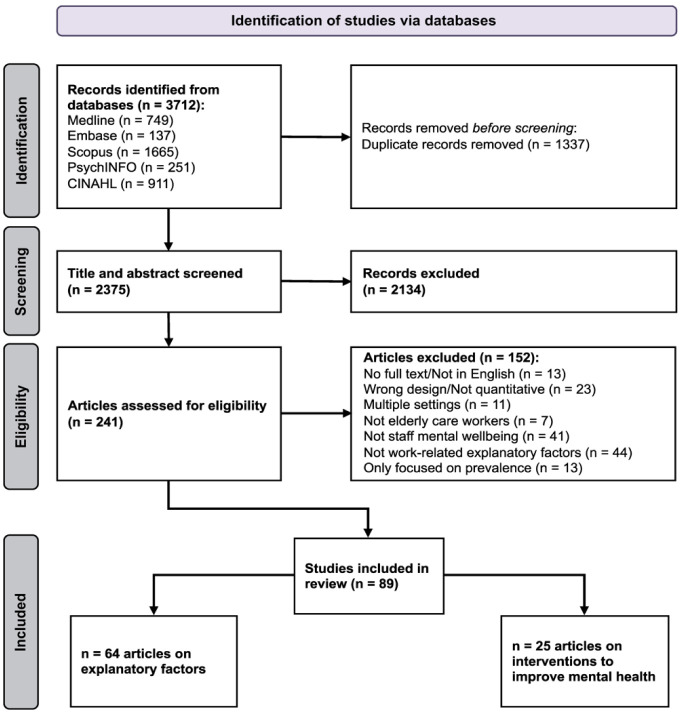
Search and review strategy.

The search retrieved a total of 3713 publications. After removing duplicates, 2375 remained for title and/or abstract review, during which 2134 publications were excluded as they did not meet the inclusion criteria. Based on the full-text assessment, a further 152 publications were excluded, resulting in 89 included studies, with 64 examining factors impacting the work-related mental wellbeing of the aged care workforce and 25 evaluating intervention studies aiming to improve staff mental wellbeing. Figure 1 demonstrates the inclusion and exclusion of papers at each stage of the screening process.

### 3.2. Summary characteristics of the included studies

A summary of the key characteristics of the included studies is presented in Table 1 (see Supplementary File 4 for included studies). Corresponding authors were spread across 24 high-income countries, with most coming from the United States (*n* = 17, 19.1%), followed by Germany (*n* = 8, 9.0%), Japan (*n* = 8, 9.0%), and Canada (*n* = 7, 7.9%). Almost half of the 89 included studies concentrated on nursing staff (*n* = 38, 42.7%), while around a quarter focused on direct care workers (*n* = 25, 28.1%), or aged care workers more broadly (i.e., direct and indirect care workers; *n* = 19, 21.3%). Few included studies concentrated specifically on personal care workers/residential care aides (*n* = 3, 3.4%) or home care workers (*n* = 2, 2.2%). The included studies were set in aged care facilities or nursing homes (*n* = 85, 95.5%), with only two studies conducted in home care (*n* = 2, 2.2%) or aged care services across home-based and aged care facilities (*n* = 2, 2.2%). Two-thirds of the studies were cross-sectional (*n* = 58, 65.1%), while one-quarter were intervention studies (*n* = 25, 28.1%); a smaller proportion were cohort studies (*n* = 7, 7.9%). All 89 studies utilized surveys for data collection, with a small number of studies also capturing qualitative data (*n* = 5, 5.6%) or incorporating additional quantitative data sources (*n* = 3, 3.4%). Over half the included studies assessed burnout as a mental health outcome (*n* = 48, 53.9%), mostly utilizing the Maslach Burnout Inventory (*n* = 36, 75.0%).

**Table 1.** Summary of key characteristics of included studies (*n* = 89).

**Classification**	**Number of studies**	**%**
**Country of the corresponding author**		
United States	17	19.1
Germany	8	9.0
Japan	8	9.0
Canada	7	7.9
Australia	6	6.74
Other	43	48.3
**Participants**		
Nursing staff	38	42.7
Direct care workers	25	28.1
Aged care workers	19	21.3
Personal care workers	3	3.4
Allied health professionals	2	2.2
Home care workers	2	2.2
**Setting**		
Aged care facilities/nursing homes	85	95.5
Aged care services	2	2.2
Home care	2	2.2
Study design*		
Cross-sectional	58	65.1
Intervention	25	28.1
Longitudinal/Cohort	8	8.9
Study methods*		
Survey	89	100
Other qualitative data	5	5.6
Other quantitative data sources	3	3.4
Mental health outcome*		
Burnout	48	53.9
Stress/Distress	32	36.0
Mental wellbeing	18	20.2
Depression	12	13.5
Anxiety	3	3.4

Note: *Does not sum to 100% owing to multiple response options; direct care workers provide direct care to older adults (e.g., nurses, medical practitioners, personal care workers, and allied health practitioners); personal care workers are direct care workers who provide non-medical assistance to older adults. Aged care workers include all direct and non-direct care roles (e.g., gardeners and cleaners).

### 3.3. Quality assessment

Of all the studies reviewed, 59 studies (66.3%) achieved a rating of high quality. Twenty-seven studies (30.3%) were evaluated as medium quality, while the remaining three were rated low quality (3.4%). The major reasons for the low-quality scores were related to not using valid and reliable exposure measurements, or not reporting inclusion criteria or potential confounding factors. Among the 25 intervention studies, all were rated as either high (*n* = 18, 72%) or medium (*n* = 7, 28%) quality, with no studies classified as low quality (See Supplementary File 4 for the quality classification of each study).

### 3.4. Factors impacting the mental wellbeing of aged care workers (n = 64 studies)

**Figure 2.**
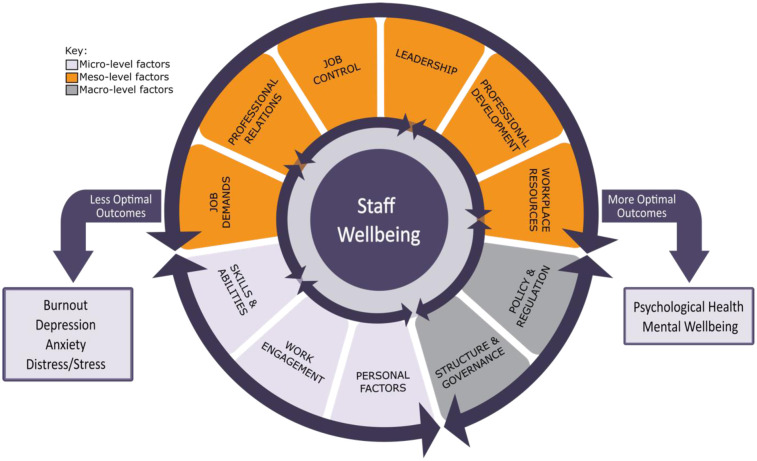
Factors Affecting Mental Wellbeing of Aged Care Workers (*n* = 64 studies). Aged care workers are at the center of the nucleus. Encircling the nucleus are factors at the micro-, meso-, and macro-levels that influence staff wellbeing, each represented by a distinct color. Each level is subdivided into themes, arranged according to the frequency of studies identified in the review. The arrows around the nucleus convey the interconnectivity of the factors affecting staff wellbeing, and the arrows around the outside convey the interconnectivity between system levels.

Based on our analysis of the 64 studies examining contributory factors, 3 themes were identified at the micro-level (personal factors; work engagement; skills and abilities), 6 themes at the meso-level (job demands; professional relations; job control; leadership; professional development; workplace resources), and 2 at the macro-level (policy and regulation; structure and governance). Figure 2 provides a visual summary of the 11 themes at the different system levels, and their corresponding sub-themes are outlined in Table 2. The results for each of the 11 themes are discussed below, with themes and sub-themes **bolded** and *italicised*, respectively.

**Table 2.** Wellbeing Themes, Subthemes, and Indicators (*n* = 64 studies).

**Theme**	**Subthemes**	**Example indicators**
**Micro-level factors**
Personal Factors	Demographics	Age, gender, and race
	Personal attitudes and values	Ageism, altruistic values
	Physical and emotional health	Self-esteem, exercise, and insomnia
	Work-life balance	Work/family conflict
	Relationships and social support	Support from family/friends
	Personal or family-related stress	Personal/family stress
Work engagement	Job satisfaction	Job satisfaction, job dissatisfaction
	Organizational commitment	Organizational identification
	Satisfaction with care quality	Care quality perception
	Satisfaction with salary	Perceived fair pay
Skills & abilities	Work competency and experience	Skills and knowledge
	Coping skills	Adaptive coping, avoidance
	Life orientation	Sense of coherence, meaningful life
**Meso-level factors**
Job demands	Physical and emotional demands	Physical demands, mental stress
	Workload	Workload stress, job-level workload
	Resident-related factors	Patient behavior, agitation/aggression
	Time pressure	Tight work schedule, time pressure
	Working hours	Working hours, shift arrangements
	Roles and responsibilities	Work seniority, occupational position
	Work stress related to pandemics	COVID cases in the workplace
Professional relations	Co-worker support	Support from co-workers
	Culture and team collaboration	Safety culture, social capital
Job control	Job autonomy/control	Job autonomy, job control
	Empowerment to modify work	Psychological empowerment self-determination
	Job complexity	Job complexity, mental regulation
Leadership	Management support	Relationship with superiors
	Leadership engagement	Participation in organizational affairs
	Leadership style	Managerial domination
Professional development	Job preparation	Lack of job preparation, job orientation
	Job training	Training
	Rewards and incentives	Opportunities for promotion
Workplace resources	Staffing and resources	Staffing/resource adequacy
	Organizational slack	Organizational slack-staff
**Macro-level factors**
Policy & regulation	National and state policies	Salary, productivity standards
	COVID-specific factors	Incoming changes during pandemic
Structure & governance	Ownership	Private/public, corporation-affiliated
	Facility size	Institution size
	Location / health region	Province / health region
	Service type	Community care, residential aged care

#### 3.4.1. Micro-level factors: Personal factors

**Personal factors** impacting the mental wellbeing of the aged care workforce were identified in almost half the 64 included studies examining factors (*n* = 29, 45.3%). The most consistent *demographic* factor affecting staff mental wellbeing was age, with older workers reporting better psychological health [35]–[38], while younger workers exhibited greater burnout [39]–[47], depression [48], and stress [49],[50]. However, the results of studies examining gender differences in mental wellbeing were less consistent. In aged care facilities in Taiwan, Lin, Chen, Huang et al. [51] identified that female nursing staff presented higher levels of depression and anxiety compared with male nursing staff. On the other hand, the study by Tanaka, Iso, and Sagari et al. [46] on aged care workers in Japan found that being male was associated with the depersonalization dimension of burnout. Only 2 studies from the United States revealed race as a significant factor, with ‘Black workers and other racial groups' displaying better mental health compared to their ‘White’ counterparts [37],[38].

Five studies revealed *personal attitudes and values* as factors of mental wellbeing. Of these, 3 studies showed that negative staff attitudes toward older adults, such as ageism and aversion to elderly patients, were associated with higher levels of stress and burnout [46],[52],[53]. Duan, Song, and Thorne et al. [54] identified that having a sense of meaning in work (i.e., personal value in work) was associated with more favourable mental health patterns. However, Eder and Meyer [55] found a more complex pattern of results; aged care nurses who held high altruistic job values and reported having a low ability to say ‘no’ when asked to work overtime faced an increased risk of burnout.

Four studies highlighted the importance of *physical and emotional health* for workers' mental wellbeing. In these studies, lack of exercise, nutrition, and sleep were identified as predictors of poor mental wellbeing [56],[57], depression [48], and stress [58]. Additionally, four studies investigating *work-life balance* found that when work demands interfered with family responsibilities, work-family conflict could arise, negatively impacting mental wellbeing [37],[38],[59] and contributing to burnout [60]. Furthermore, *social support* from family and friends was identified as a protective factor against psychological distress [61],[62] and burnout [44]. Conversely, *personal or family-related stress* was reported as a risk factor for increased work-related stress [63] and burnout [44].

*Relationships*, including familial and marital status, were also found to play a significant role in mental wellbeing. One study involving Japanese and Korean aged care workers found that unmarried Japanese care workers exhibited higher levels of burnout [45]; however, marital status did not significantly impact burnout levels among Korean care workers. Another study, investigating the mental wellbeing of nursing staff working in aged care facilities during the COVID-19 pandemic, reported that nurses who were married presented higher depression and anxiety than those who were not married [51]. In this case, concerns over the increased risk of spreading COVID-19 to family members may have resulted in married staff exhibiting higher levels of depression and anxiety [51].

#### 3.4.2. Micro-level factors: Work engagement

Factors related to **work engagement** (i.e., how people feel about their jobs) were identified in almost a quarter of the 64 studies (*n* = 15, 23.4%). Six studies reported a negative relationship between *job satisfaction* and burnout [45],[64]–[66], job stress [63], and depressive symptoms [67]. Work dissatisfaction was found to characterize a higher stress profile [53], while having an ‘engaged’ work profile [54] was associated with low levels of burnout and high job satisfaction among care aides employed in Canadian care homes. In 3 studies, staff *organizational commitment* was negatively related to burnout [47],[68] and sleep disturbances [69], and positively related to psychological wellbeing [69]. How staff perceive or feel *satisfied with the quality of care* at work was also identified as a predictor of burnout [42],[70],[71] and psychological stress [72]. For example, a study of nurses from German aged care homes found that those who became or continued to be dissatisfied with the quality of care in their facility over a two-year period experienced higher levels of burnout compared to those who remained satisfied with care quality [71].

A further 2 studies suggested that *satisfaction with salary* contributed to mental wellbeing. In a study of aged care workers from Austria, Jenull and Wiedermann [53] found that workers who perceived their financial position as adequate were more compatible with a low-stress profile. Similarly, perceived fair pay was inversely related to burnout in a study of direct care workers in the United States [47].

#### 3.4.3. Micro-level factors: Skills and abilities

In 14 studies (21.9%), factors related to **skills and abilities** were identified. Among these, 8 studies entailed factors related to *work competency and experience*. The findings generally suggested that higher levels of work competency (e.g., belief in one's skills and knowledge about aging), as well as greater work experience, were linked to lower levels of burnout [41],[52],[54],[73] and psychological stress [74]. However, three studies identified that longer tenure in the aged care sector was associated with increased burnout [47],[75] as well as depression and anxiety [51]. For example, Lin, Chen, Huang et al. [51] reported that nursing staff in Taiwan with more than 10 years of aged care experience exhibited higher levels of depression and anxiety. These authors attributed these findings to increased job demands and responsibilities often assigned to staff with greater experience [51].

The examination of *coping skills* across 5 studies consistently demonstrated that the use of adaptive coping strategies (e.g., active planning, positive reframing, and resilience) was associated with enhanced wellbeing [76] and lower levels of burnout [73] and depression [67]. Conversely, maladaptive coping strategies (e.g., experiential avoidance, negative affectivity) were associated with higher levels of burnout [77],[78] and depression [77].

Three additional studies examined aspects of *life orientation*; two of which assessed sense of coherence, defined as the ability to understand situations and effectively utilize coping strategies, and its impact on aged care staff wellbeing. These studies showed that a higher sense of coherence was protective against burnout [79] and psychological stress [62]. Another study entailed staff perceptions of life satisfaction (i.e., having a meaningful life) and showed that lower levels of life satisfaction were associated with burnout and depression [77].

#### 3.4.4. Meso-level factors: Job demands

Factors related to **job demands** were identified in half of the 64 studies (*n* = 32, 50.0%). Twelve studies emphasized the negative effects of the *physical and emotional demands* of working in the aged care industry on psychological stress [36],[49],[57]–[59],[61],[80],[81], mental wellbeing [36],[37],[57],[76], depression [49],[61],[82], burnout [44],[78], and anxiety [49]. Examples of physical demands of aged care work included frequent lifting and transferring of residents, and emotional demands included responding to the emotional needs of residents and families suffering and dealing with death and dying. A further 6 studies entailed the *workload* of the aged care workforce found that a heavy perceived workload (i.e., having too much work to do) was associated with increased burnout [40],[41],[64],[83] and psychological stress [40],[50],[62].

Job demands in aged care can also arise from *resident-related factors*. Four studies revealed that challenges related to resident behavior, such as verbal abuse (e.g., yelling and screaming) and physical aggression (e.g., being bitten and pushed), were linked to increased psychological stress [72],[84] and burnout [54],[65] among aged care workers. Three studies emphasized *time pressure*, such as being pressed for time or having to rush care because of high workload, as contributors to burnout [54],[85] and diminished mental wellbeing [56].

Several other studies entailed position-related factors, including *working hours* and *roles and responsibilities*. Three studies showed that longer working hours (specifically, exceeding 45 hours per week) and the necessity of working overtime were linked to increased psychological stress [50], higher rates of depression [48], and overall poorer mental wellbeing. Two studies showed that roles with increased responsibility, such as being a department head or registered nurse, were associated with heightened levels of psychological stress [63] and poorer mental wellbeing [35]. Another study from Germany showed that geriatric nurses working in aged care homes experienced higher levels of psychological distress and depression compared to other aged care nurses [49].

*Work stress related to the COVID-19 pandemic* emerged as a notable factor intensifying job demands in aged care settings in more recent studies [39],[49],[79],[86],[87]. One study showed that increased working hours and perceived health deterioration during the pandemic were associated with heightened burnout [79]. Another study revealed ‘infection control fatigue’, driven by the complexity of nursing duties, staff shortages, and conflicts arising from the uncertainty of the pandemic, as a contributor to burnout [86]. Fear of COVID-19 was also correlated with severe burnout among aged care staff [70].

#### 3.4.5. Meso-level factors: Professional relations

**Professional relations** and their effect on the mental wellbeing of the aged care workforce were examined in 18 of the 64 studies (28.1%). Of these, 11 studies revealed the importance of *co-worker support*, with collegial and supportive workplace relationships serving as a buffer against burnout [44],[60],[66],[73],[79],[88],[89] and psychological stress [39],[59],[61], while positively impacting mental wellbeing [36],[37]. One study further showed that when both co-worker support and job control were high, depression was lower than when individuals experienced only high job control [61]. Additional findings regarding job control are discussed below.

The significance of a positive *culture and team collaboration* was also highlighted in seven studies. Culture, often characterized as “the way we do things around here” [54], encompassed indicators related to a sense of community at work, collaboration among team members, and a positive safety culture. Across these studies, culture and collaboration were consistently negatively associated with psychological stress [49],[57],[72], burnout [40],[42],[54], depression [49],[90], and anxiety [49], while being positively associated with mental wellbeing [57].

#### 3.4.6. Meso-level factors: Job control

Significant factors related to **job control** were identified in a quarter of the studies (*n* = 16, 25.0%). *Job autonomy/control*, defined as having control over work tasks and schedules, emerged as a predictor in 10 studies, which identified that higher levels of autonomy and control were linked to improved mental wellbeing [35]–[37],[56], and lower levels of burnout [40],[45],[66] and psychological stress [36],[40],[61]. The implication of these studies indicates that increased job autonomy or control can help mitigate the negative mental health effects associated with job demands and work-related stressors [45],[66]. In support of this perspective, Elovainio, Heponiemi, and Kuusio et al. [81] examined the buffering effects of job control against job demands on psychological distress. However, Kubicek, Korunka, and Tement [85] identified a more complex pattern of results, with a curvilinear rather than a linear relationship identified between job control and burnout among nursing staff in aged care facilities. Specifically, job control was beneficial only up to a certain threshold; beyond this point, higher levels of job control were associated with increased burnout [85].

In a similar way, findings regarding *empowerment to modify work* were not straightforward. One study of personal care workers in Canada showed that increased self-determination at work was associated with a more ‘engaged’ work profile and low levels of burnout [54]. Another study of burnout in United States nursing homes reported that aged care workers who felt more empowered to modify their work were more likely to report personal accomplishment [47]. In another study of European direct care workers, while a positive linear relationship between ‘job crafting’ (defined as involving three dimensions: Task crafting, relational crafting and cognitive crafting) and mental wellbeing was identified in the Spanish sample, the Swedish sample showed that both low and high levels of job crafting were associated with lower mental wellbeing, and medium levels of job crafting were linked to optimal mental wellbeing [91].

The findings across a further 3 studies suggested that aged care workers whose work is characterized by *job complexity* were less likely to report burnout [41],[75] or depression [92]. Aspects contributing to job complexity in aged care include, for example, the need to balance different expectations (e.g., of family members, care recipients, and professional standards) or goals (e.g., cost efficiency, detailed documentation, and providing activating/stimulating care) [75]. Job complexity also correlated with increased job responsibility, particularly among senior staff expected to manage more challenging tasks [41].

#### 3.4.7. Meso-level factors: Leadership

Factors related to **leadership** were found to impact the mental wellbeing of the aged care workforce in a quarter of the studies (*n* = 16, 25.0%). *Management support* was identified in 13 of these studies and was protective against burnout [40],[44],[47],[60],[64],[73],[88],[89], psychological stress [40],[57],[59],[61],[72],[73],[80], and mental wellbeing [57]. For example, Blanco-Donoso, Moreno-Jiménez, and Amutio et al. [80] identified that the presence of sufficient supervisor support moderated the impact of work stressors on traumatic stress during the COVID-19 pandemic among nursing home workers in Spain.

Four studies revealed *leadership engagement* as a positive factor on staff mental wellbeing [42],[54],[57],[64]. Establishing leadership engagement through management commitment, management priority, as well as organizational communication and participation, was found to have a negative association with compassion fatigue, a mental wellbeing outcome related to burnout [42]. Conversely, another study indicated that managers not engaging their staff in decision-making on issues affecting them were found to negatively impact mental wellbeing [57]. Furthermore, Muntaner, Ng, Prins et al. [82] identified that a *leadership style* of managerial domination (i.e., bureaucratic styles of management) had a detrimental effect on depression.

#### 3.4.8. Meso-level factors: Professional development

Factors related to **professional development** were identified in 9 of the 64 studies (14.1%). Three studies reported findings related to *job preparation*, which identified that inadequate job orientation had a negative effect on engagement [54], perceived competence [43], and burnout [40],[43],[54]. Additionally, *job training* was a relevant professional development factor that positively influenced aged care workers' mental wellbeing [42],[47],[93]. Jameson and Parkinson [42] found a negative association between the perceived level of training of personal care attendants and compassion fatigue.

Wu, Yamaguchi, and and Greiner [62] analyzed workers in aged care facilities in Japan and found that the provision of *rewards and incentives* was associated with better mental health scores. In another study from the United States, workers in nursing homes that did not provide seniority-based increases in wages were more likely to suffer from depressive disorder than in facilities that offered wage increases based on seniority [82].

#### 3.4.9. Meso-level factors: Workplace resources

**Workplace resources** related to meso-level factors were identified in eight studies (12.5%). Six studies revealed *staffing and resources* as factors important for aged care workers' mental wellbeing. In these studies, staffing and resource adequacy were negatively associated with burnout [47],[79],[89] and stress [72],[80],[81]. In addition, a lack of staff and personal protective equipment (PPE) were associated with secondary traumatic stress among workers, regardless of whether they had contact with COVID-19 patients [80]. Staffing level was associated with perceived stress, psychological distress, and sleeping problems, indicating that lower staffing levels were associated with an increased risk of psychological distress [81].

Two studies highlighted the importance of *organizational slack* for workers' mental wellbeing [54],[65]. For example, Chamberlain, Gruneir, Hoben et al. [65] identified that fewer staffing resources and insufficient space to discuss resident care needs (defined as ‘organizational slack-staff’ and ‘organizational slack-space’, respectively) were associated with increased staff burnout.

#### 3.4.10. Macro-level factors: Policy and regulation

At the macro-level, factors related to **policy and regulation** were identified in 11 studies (17.2%). In terms of *national and state policies*, increased staff psychological distress was associated with lower staff-to-resident ratios [81]. Another study showed that increased productivity standards (i.e., billable staff time) were related to burnout in aged care settings in the United States [83]. Moreover, professionals with increased productivity standards reported greater pressure to participate in unethical practices, such as prolonging patient caseloads, altering clock-in times, falsifying documentation, and modifying billing codes [83]. Kim and Choi [94] found that an enhanced compensation system (including salary, promotions, bonuses, and welfare allowances) improved staff retention and reduced burnout risk in Japan. Similarly, several other studies reported the importance of fair pay for improving mental wellbeing [36] and reducing psychological stress [53] and burnout [12],[41]. Further, performance-based wage systems have been associated with increased depression and job insecurity [82]. Additional global pressures during the COVID-19 pandemic, such as compliance with shifting regulations (e.g., frequent PCR tests, changes in working hours, and fluctuating staff ratios), intensified psychological stress [62] and burnout [79] among aged care workers.

#### 3.4.11. Macro-level factors: Structure and governance

Factors related to **structure and governance** were identified in 9 studies (14.1%). The most prevalent factor within this domain was *ownership*, where staff in for-profit nursing homes were more likely to experience adverse mental health outcomes, including higher levels of anxiety, depression, and burnout, compared to those in not-for-profit or public facilities [51]–[53],[82]. One study linked private ownership to increased depersonalization and overall burnout [52], while another attributed severe depression and anxiety among staff in private and foundation-affiliated facilities to fewer medical and pandemic prevention resources, compared to public facilities [51]. Furthermore, burnout risk was shown to decrease in facilities with full-time psychologists, emphasizing the potential mitigating effects of workplace resources [53].

*Facility size* was examined in three studies, yielding mixed findings. One study found that smaller institutions were associated with better health-related quality of life among staff [36]. Conversely, another study reported that staff in larger facilities (e.g., more than 100 residents) experienced less stress than those in smaller care homes [49]. This study suggested that the relationship between facility size and mental health outcomes was influenced by contextual factors, such as the presence of infection control protocols, highlighting that larger facilities were better equipped to implement measures that may mitigate staff stress [49]. A third study, however, reported no significant relationship between facility size and staff mental health outcomes [95].

Two studies investigated *facility location* as a factor [43],[53]. In one study, mental health and burnout outcomes varied greatly across health regions in Canada, with some areas reporting better outcomes than others [43]. Similarly, in Austria, nurses working in Vienna were found to have poorer mental health outcomes, largely due to challenges such as language barriers, which contributed to elevated work-related stress [53]. Finally, *service type* was identified as a relevant factor by Jameson and Parkinson [42], highlighting that aged care workers in RACFs experienced higher levels of compassion fatigue than those in community care settings.

### 3.5. Interventions to improve the mental wellbeing of aged care workers (n = 25)

We identified 25 intervention studies aimed at improving the mental wellbeing of the aged care workforce. The characteristics of these studies are presented in Supplementary File 4. All studies entailed a longitudinal study design, including eight RCTs, 7 non-randomized controlled trials, 9 longitudinal studies without a control group, and 1 case-control study. Intervention durations ranged from a single 90-minute education session [96] to ongoing implementation studies lasting over 12 months [97]. Most interventions were assessed in the medium term (1–11 months: *n* = 14, 56.0%), with fewer studies conducting short-term (0–1 month: *n* = 7, 28.0%) or long-term (≥12 months: *n* = 4, 16.0%) follow-ups. Over half of the intervention studies evaluated burnout (*n* = 14, 56.0%) and or stress/distress (*n* = 13, 52.0%) as mental health outcomes, while fewer assessed mental wellbeing (*n* = 6, 24.0%), depression (*n* = 3, 12.0%) or anxiety (*n* = 1, 4.0%).

Almost all interventions targeted the micro-level (individual staff; *n* = 24, 96%), and included: Relaxation training (e.g., Tai Chi [98], yoga [99], coherent breathing [100]); emotion regulation training (e.g., mindfulness [101],[102], cognitive behavior therapy [103], the butterfly hug of bilateral stimulation [104], and psychological first aid [105]); and behavioral management education, primarily for managing challenging behaviors in residents with dementia [97],[106],[107]. At the meso-level, there were 13 studies, which focused on improving staff-resident interactions through patient-centered care approaches [97],[106],[108],[109]; optimizing work processes (e.g., teamwork, balancing workload, and time management) [97],[106],[108]–[111]; improving professional relations [110],[112]–[114]; and decision-making related to infection control strategies during pandemics [97],[106],[107],[109],[111]–[113],[115],[116]. One study entailed a technology-based medication and care support system [39] aimed at reducing staff stress and medication errors in nursing homes. While no interventions were directed at the macro-level, one study encompassed the implementation of Swedish national guidelines of person-centered care in residential aged care settings and their impact on staff stress [109].

Nearly two thirds (*n* = 16, 64.0%) of the intervention studies reported improvements in staff mental wellbeing post-intervention, including significant reductions in burnout [96],[101],[106], stress/distress [39],[96],[98],[100],[106],[109], depression [100], and anxiety [100],[117], as well as increases in overall mental wellbeing [98],[103],[104],[114],[118]. Of these, 11 studies reported improved mental wellbeing across all measured outcomes, and five studies reported a mix of null and positive results. One example of a study that demonstrated positive results across all measured mental wellbeing outcomes involved the evaluation of a 90-minute self-care skills training program, which was supplemented by a self-care skills toolbox that participants could continue to access and use [96]. Using a pre-post-follow-up design, significant reductions in nurse burnout and stress were identified, alongside a 43% increase in staff retention after one month [96]. In another example, Barbosa, Nolan, and Sousa et al. [106] employed a pre-post-test control group experimental design to evaluate weekly psychoeducational intervention sessions grounded in person-centered care. These sessions improved worker-resident interactions, decreased negative communicative behaviors, reduced resident agitation and aggression, and enhanced staff coping strategies, resulting in reduced burnout and improved communicative behaviors, such as involvement.

The remaining one-third of intervention studies (*n* = 9, 36.0%) reported no significant effects [97],[105],[107],[108],[110],[113],[115],[116],[119]. For example, while Gillis, van Diermen, and Lips et al. [113] found that an eight-session person-centered ‘need-based care’ intervention improved staff competence, professionalism, and relationship-building, but it did not significantly reduce burnout. In another study on Psychological First Aid (PFA) [120], a WHO-developed intervention was designed to alleviate the effects of crises, chronic stress, and trauma. In this study, despite PFA training being identified as a predictor of coping efficacy, no significant improvements in staff stress, connectedness, or sense of accomplishment were found [105].

## 4. Discussion

In recent years, there has been increasing recognition of the importance of prioritizing the mental wellbeing of care workers. This review contributes to the literature by synthesizing key factors associated with the mental wellbeing of the aged care workforce across multiple system levels and examining the interventions implemented to address these challenges.

### 4.1. Multi-level model of aged care worker mental wellbeing

From our synthesis, a multi-level model of factors affecting aged care worker mental wellbeing was developed (Figure 1), comprising 11 themes and 39 sub-themes; 3 micro-level themes (personal factors; work engagement; skills and abilities), 6 meso-level themes (job demands; professional relations; job control; leadership; professional development; workplace resources), and 2 macro-level themes (policy and regulation; structure and governance). The model, inspired by the NAM Conceptual Model of Factors Affecting Clinician Wellbeing and Resilience [33], is grounded in empirical evidence from 64 studies set within the aged care context reporting factors of staff mental wellbeing.

Consistent with the original NAM model [33], our goal was not to establish a hierarchy but to provide a framework for identifying potential leverage points and generating solutions at the individual, organizational, and broader systems levels. The interconnectivity of the factors, represented by the arrows around the nucleus in Figure 1, must be emphasized. Further research is needed to explore how these interconnected factors interact and influence outcomes, and how this can inform the design of effective interventions. For example, national aged care salary policies at the macro-level foreseeably influence organizational resources, job demands, opportunities for career development, and job stability at the meso-level, which, in turn, affect staff satisfaction with salary and overall job satisfaction at the micro-level. However, these interconnections have been minimally studied to date [121]. Indeed, recent policy changes in Australia, which included a 15% increase in award rates for aged care workers in 2023, with an additional 2.3% to 13.5% increase due in 2025, provides a unique opportunity to examine the impact of salary increases on meso- and micro-level factors, as well as on staff retention [122].

That being said, the interconnectivity between several meso-level factors has been explored in the broader healthcare literature. For example, the Job Demand-Control-Support (JDCS) model posits that high job demands, combined with low control over work and limited support from colleagues or supervisors, are associated with poorer mental wellbeing, lower job satisfaction, and increased turnover intentions and burnout [123]. However, this model has yet to be thoroughly examined within the specific context of aged care, and there is limited understanding of how it can be applied to inform the design of targeted interventions.

### 4.2. Key factors of aged care worker mental wellbeing

Consistent with previous research [12], at the micro-level, several personal factors such as age, gender, and personal attitudes and values were identified. For example, younger workers and those with negative attitudes towards older adults reported higher levels of stress and burnout [41]–[43],[53]. This aligns with previous studies from healthcare settings that indicate that younger healthcare workers are more susceptible to burnout due to less experience and inadequate coping mechanisms [124],[125]. Work experience and adaptive coping skills were identified as key factors for aged care staff wellbeing, aligning with previous research emphasizing the importance of competence and coping mechanisms in mitigating stress and burnout among healthcare professionals [124].

Meso-level factors, including job demands, professional relations, job control, leadership, professional development, and workplace resources, were also associated with aged care staff wellbeing. High workloads, time pressures, and emotional demands were consistently associated with poorer mental health outcomes. Effective leadership and supportive management were protective factors, reducing burnout and psychological distress among workers. However, the findings regarding job control were less clear. While there was generally evidence to suggest that increased job control was associated with improved mental wellbeing, a more complex pattern also emerged. In support of the ‘Vitamin model’, Kubicek, Korunka, and Tement [85] found that both low and high levels of job control were linked to work-related wellbeing in a longitudinal study of aged care workers. The authors reasoned that for aged care workers, higher levels of job control often come with increased responsibility and demands, which may offset the potential benefits of control. Further research is needed to examine the interaction between job control and other factors [85], such as coworker and managerial support, workplace culture, and team collaboration to gain a more detailed understanding of these dynamics within the aged care sector specifically.

At the macro-level, structural factors such as ownership and service type influenced the work environment and staff mental wellbeing. For-profit nursing homes reported higher levels of anxiety, depression, and burnout compared to public or not-for-profit facilities, attributed to fewer resources and poorer working conditions [14]. Policies promoting adequate staffing, fair compensation, and workplace resources, such as full-time psychologists, were shown to reduce burnout, while factors like lower staff-to-resident ratios and performance-based compensation linked to seniority contributed to higher burnout and depression [126].

### 4.3. Interventions

We identified 25 intervention studies, primarily focused on micro-level approaches, including relaxation, emotion regulation, and behavioral management education training. These interventions largely align with micro-level factors related to improving skills and abilities. Some meso-level interventions were also identified that targeted training to optimize work processes, decision-making, teamwork, and staff interactions with residents through patient-centered care approaches, but no macro-level interventions were identified. The meso-level interventions were largely aligned in focus to the meso-level factors (job demands, job control, professional relations, leadership, and workplace resources) identified.

Many interventions (64%) showed immediate wellbeing benefits, including reduced stress, burnout, and emotional exhaustion, consistent with prior research on the effectiveness of mindfulness and emotion regulation techniques in reducing burnout among aged care workers [127],[128]. However, one-third of the interventions also reported no significant impact, aligning with previous broader healthcare research [129],[130], highlighting the uncertainty of long-term mental wellbeing benefits with diminishing effects on job satisfaction and burnout over time.

The promotion of mental wellbeing through interventions requires time and efforts from all involved; as such, the prioritization of “valuable” interventions is therefore of utmost importance for already strained workers in elderly care. If interventions are not prioritised based on their demonstrated value, the introduced interventions risk being perceived as an extra burden instead of as an opportunity for improvements to mental wellbeing [131]. Greiner, Leduc, and O'Brien et al. [132] also identified implementation challenges for meso- and macro-level interventions, including low uptake and limited broader outcomes, like job satisfaction or engagement. Despite the described challenges of uptake and ownership of meso- and macro-interventions, interventions targeting the organizational- and system-level have been found to reveal more sustainable and positive impact than interventions aimed at the micro-level [133]. These findings are of key importance, particularly for leaders in aged care facilities seeking to improve staff wellbeing. Combining the role of leadership and meso- and macro-level interventions stands out as an impactful way for long-term positive effects. Additionally, Fox, Johnson, and Berkman et al. [134] in their review of organizational workplace interventions identified that interventions that create opportunities for employees to participate through feedback and process modification are particularly effective. Further research is needed to better understand the complexity of factors affecting staff mental wellbeing, including process evaluations that explore the conditions under which specific interventions can lead to sustained, positive change [134].

### 4.4. Strengths and limitations

Our strengths include the expansive search strategy, with five databases, the inclusion of multiple wellbeing indicators, and the combination of inductive and deductive data analysis and synthesis, drawing on the NAM Conceptual Model for Clinician Wellbeing and Resilience. Using a data-driven convergent synthesis approach [135], we transformed data from quantitative studies into common factors and themes.

As to limitations, focusing solely on quantitative studies, we may have overlooked important factors and interventions identified in qualitative research. As always, restricting to English-language and published journal articles could have excluded relevant findings. We identified relatively few studies on home care, possibly due to limitations in our search strategy related to home care and home care aides. While our search strategy was comprehensive, a more targeted search focused on home care studies may be warranted. Our data extraction was restricted to significant factors reported in the included studies, excluding non-significant findings. Thus, we may have under-identified some important factors and negative mental wellbeing consequences. Further, almost two-thirds of the studies were cross-sectional, and while significant associations were found, this does not imply causation. Owing to the complexity of the results, assigning a definitive positive or negative sign to indicate the association between each indicator and staff mental wellbeing was often not straightforward. As such, the results are not presented this way; instead, we recommend interpreting the findings comprehensively by reviewing the results in full. We generalized factors between countries and health systems; however, comparing factors across studies is inherently challenging, as the socio-cultural context and the roles and responsibilities of aged care workers differ significantly between countries [15]. For this reason, we focused on identifying common factors across studies.

## 5. Conclusions

Improving aged care workers' mental wellbeing requires addressing factors across system levels. Our new evidence-based model depicts domains at multiple system levels (micro, meso, and macro) to guide organizations in developing targeted strategies to promote the mental wellbeing of aged care workers and improve resident care. While individual-focused interventions have shown benefits, system-level strategies are key to improving mental wellbeing and achieving sustainable change across the board. We conclude with some key recommendations for aged care organizations in Box 1.

Box 1. **Key recommendations for aged care leaders, policy, and practice.**

**Prioritize staff mental wellbeing**

- Senior leadership should recognize and prioritize the importance of staff mental wellbeing as a core organizational value.
- Implement a comprehensive mental health policy, including a policy that promotes greater flexibility and work-life integration to support staff balance and reduce stress.
- Regularly assess staff mental wellbeing using standardized instruments in addition to other key priority areas, such as job satisfaction and turnover.
- Involve all staff members in the assessment process and utilize instruments with national benchmark data to aid in the interpretation and comparison of results.
- Aggregate data to a facility-level to enable leadership to direct attention and resources to where they are most needed.

**Implement targeted interventions.**

- Senior leadership needs to understand the micro-, meso-, and macro-level factors influencing staff mental wellbeing, including the interdependencies between factors, as well as the effective tools and interventions that can address these factors.
- Senior leaders can use the model developed in this study to identify leverage points and prioritize specific areas to focus the intervention.
- Keep the intervention targeted; interventions that focus on one or two changes and create opportunities for workers to participate through feedback and process modifications seem to be particularly effective.
- Senior leaders must be provided with resources, skills, and structures to tailor, implement, and evaluate targeted interventions.

**Cultivate a collaborative culture**

- Recognition that leadership support is crucial in staff mental wellbeing.
- Encourage a supportive, collaborative culture; forging connections among staff using deliberate strategies.

**Provide resources to promote self-care**

- Provide aged care staff with tools and training for relaxation and self-regulation, and behavioral management; identified as important for promoting individual resilience and mental wellbeing.

## Use of AI tools declaration

The authors declare they have not used Artificial Intelligence (AI) tools in the creation of this article.


