# Supplementary material for: Supporting the mental wellbeing of aged care workers: A systematic review of factors and interventions
Source: AIMS Public Health. 2025 Jun 17;12(2):600–31. doi: 10.3934/publichealth.2025032 (PMC12277774; doi:10.3934/publichealth.2025032)
Supplement: Supplementary file 1 [file publichealth-12-02-032-s001.pdf]

Public Health

AIMS Public Health, 12(2): 600–631.
DOI: 10.3934/publichealth.2025032
Received: 24 December 2024
Revised: 02 May 2025
Accepted: 20 May 2025
Published: 17 June 2025

https://www.aimspress.com/journal/aimsph

Review

Supporting the mental wellbeing of aged care workers: A systematic review of factors and interventions

Louise A Ellis[1,2,*], Tanja Schroeder[1], Maree Saba[1], Kate Churruca[1], Janet C Long[1], Annie Haver[3,4], Kristin Akerjordet[4], Kristiana Ludlow[1,5], Inger Johanne Bergerød[4], Sini Nevantaus[6], Jan-Willem Weenink[7], Zoe Gonzales[1], Samantha Spanos[1], Hilda Bø Lyng[3], Cecilie Haraldseid-Driftland[4], Daniel Adrian Lungu[4], Malin Knutsen Glette[2,8], Mari Lahti[6], Florin Tibu[9], Andreas Chatzittofis[10], Juana Maria Delgado-Saborit[11], Eila Kankaanpää[12], Viviana Wuthrich[2], Robyn Clay-Williams[1], Jeffrey Braithwaite[1,4] and Siri Wiig[1,4]

[1] Centre for Healthcare Resilience and Implementation Science, Australian Institute of Health Innovation, Macquarie University, Australia
[2] Lifespan Health and Wellbeing Research Centre, School of Psychological Sciences, Macquarie University, Sydney
[3] NHS-Department of Leadership and Service Innovation, Faculty of Social Sciences, University of Stavanger, Norway
[4] SHARE - Centre for Resilience in Healthcare, Faculty of Health Sciences, University of Stavanger, Norway
[5] Centre for Health Services Research, the University of Queensland, Australia
[6] Faculty of Health and Well-Being, Turku University of Applied Sciences, Finland
[7] Erasmus School of Health Policy & Management, Erasmus University Rotterdam, Netherlands
[8] Faculty of Health, Western Norway University of Applied Sciences, Norway
[9] Faculty of Medicine and Biological Sciences, Ştefan cel Mare University of Suceava, Romania
[10] Medical School, University of Cyprus, Nicosia, Cyprus
[11] Department of Medicine, Faculty of Health Sciences, Universitat Jaume I, Spain
[12] Department of Health and Social Management, University of Eastern Finland, Finland

* **Correspondence:** Email: louise.ellis@mq.edu.au; Tel: +61298502484.

Supplementary File 1. Preferred reporting items for systematic reviews checklist.

Section and Topic	Item #	Checklist item	Location where item is reported
**Title**			
Title	1	Identify the report as a systematic review.	p. 1
**Abstract**			
Abstract	2	See the PRISMA 2020 for Abstracts checklist.	p. 3/ Appendix 1
**Introduction**			
Rationale	3	Describe the rationale for the review in the context of existing knowledge.	p. 6
Objectives	4	Provide an explicit statement of the objective(s) or question(s) the review addresses.	p. 6
**Methods**			
Eligibility criteria	5	Specify the inclusion and exclusion criteria for the review and how studies were grouped for the syntheses.	p. 7
Information sources	6	Specify all databases, registers, websites, organisations, reference lists and other sources searched or consulted to identify studies. Specify the date when each source was last searched or consulted.	p. 7
Search strategy	7	Present the full search strategies for all databases, registers and websites, including any filters and limits used.	p.7/ Appendix 2
Selection process	8	Specify the methods used to decide whether a study met the inclusion criteria of the review, including how many reviewers screened each record and each report retrieved, whether they worked independently, and if applicable, details of automation tools used in the process.	p. 7/8
Data collection process	9	Specify the methods used to collect data from reports, including how many reviewers collected data from each report, whether they worked independently, any processes for obtaining or confirming data from study investigators, and if applicable, details of automation tools used in the process.	N/A
Data items	10a	List and define all outcomes for which data were sought. Specify whether all results that were compatible with each outcome domain in each study were sought (e.g. for all measures, time points, analyses), and if not, the methods used to decide which results to collect.	p. 8–9 / Appendix 3
	10b	List and define all other variables for which data were sought (e.g. participant and intervention characteristics, funding sources). Describe any assumptions made about any missing or unclear information.	N/A
Study risk of bias	11	Specify the methods used to assess risk of bias in the	p. 8–9

assessment		included studies, including details of the tool(s) used, how many reviewers assessed each study and whether they worked independently, and if applicable, details of automation tools used in the process.	
Effect measures	12	Specify for each outcome the effect measure(s) (e.g. risk ratio, mean difference) used in the synthesis or presentation of results.	p. 9
Synthesis methods	13a	Describe the processes used to decide which studies were eligible for each synthesis (e.g. tabulating the study intervention characteristics and comparing against the planned groups for each synthesis (item #5)).	p. 8/9
	13b	Describe any methods required to prepare the data for presentation or synthesis, such as handling of missing summary statistics, or data conversions.	p. 8/9
	13c	Describe any methods used to tabulate or visually display results of individual studies and syntheses.	p. 8/9
	13d	Describe any methods used to synthesize results and provide a rationale for the choice(s). If meta-analysis was performed, describe the model(s), method(s) to identify the presence and extent of statistical heterogeneity, and software package(s) used.	p. 8
	13e	Describe any methods used to explore possible causes of heterogeneity among study results (e.g. subgroup analysis, meta-regression).	p. 9
	13f	Describe any sensitivity analyses conducted to assess robustness of the synthesized results.	N/A
Reporting bias assessment	14	Describe any methods used to assess risk of bias due to missing results in a synthesis (arising from reporting biases).	p. 9
Certainty assessment	15	Describe any methods used to assess certainty (or confidence) in the body of evidence for an outcome.	p. 9
**Results**			
Study selection	16a	Describe the results of the search and selection process, from the number of records identified in the search to the number of studies included in the review, ideally using a flow diagram.	p. 9–10
	16b	Cite studies that might appear to meet the inclusion criteria, but which were excluded, and explain why they were excluded.	p. 10–11
Study characteristics	17	Cite each included study and present its characteristics.	Appendix 4
Risk of bias in studies	18	Present assessments of risk of bias for each included study.	p. 11
Results of individual	19	For all outcomes, present, for each study: (a) summary	p. 12–13

studies		statistics for each group (where appropriate) and (b) an effect estimate and its precision (e.g. confidence/credible interval), ideally using structured tables or plots.	
Results of syntheses	20a	For each synthesis, briefly summarise the characteristics and risk of bias among contributing studies.	p. 10–22
	20b	Present results of all statistical syntheses conducted. If meta-analysis was done, present for each the summary estimate and its precision (e.g. confidence/credible interval) and measures of statistical heterogeneity. If comparing groups, describe the direction of the effect.	N/A
	20c	Present results of all investigations of possible causes of heterogeneity among study results.	N/A
	20d	Present results of all sensitivity analyses conducted to assess the robustness of the synthesized results.	N/A
Reporting biases	21	Present assessments of risk of bias due to missing results (arising from reporting biases) for each synthesis assessed.	p. 11
Certainty of evidence	22	Present assessments of certainty (or confidence) in the body of evidence for each outcome assessed.	N/A
**Discussion**			
Discussion	23a	Provide a general interpretation of the results in the context of other evidence.	p. 22–26
	23b	Discuss any limitations of the evidence included in the review.	p. 25–26
	23c	Discuss any limitations of the review processes used.	p. 25–26
	23d	Discuss implications of the results for practice, policy, and future research.	p. 27
**Other information**			
Registration and protocol	24a	Provide registration information for the review, including register name and registration number, or state that the review was not registered.	p. 7
	24b	Indicate where the review protocol can be accessed, or state that a protocol was not prepared.	p. 7
	24c	Describe and explain any amendments to information provided at registration or in the protocol.	N/A
Support	25	Describe sources of financial or non-financial support for the review, and the role of the funders or sponsors in the review.	p. 28
Competing interests	26	Declare any competing interests of review authors.	p. 28
Availability of data, code and other materials	27	Report which of the following are publicly available and where they can be found: template data collection forms; data extracted from included studies; data used for all analyses; analytic code; any other materials used in the review.	N/A

Supplementary File 2. Search strategy.

Medline:

1 geriatrics/ or assisted living facilities/ or homes for the aged/ or nursing homes/ or intermediate care facilities/ or skilled nursing facilities/ or Long-Term Care/
2 (Aged care or elderly care or residential care or residential facilit* or long-term care or nursing care facilit* or old age home* or nursing home* or assisted living facilit* or home* for the aged or housing for the elderly or residential aged care or residential aged care facilit*).ti,ab.
3 or/1–2
4 stress, psychological/ or burnout, psychological/ or Psychological Well-Being/
5 ((mental or emotional or psychological) adj1 (wellbeing or health or wellness)).ti,ab
6 (mental health or wellbeing or burnout or anxiety or stress or distress or fatigue or depression).ti,ab.
7 or/4–6
8 health personnel/ or nurses/
9 (Health personnel or health* workers* or nurses or care worker or professional carer*).ti,ab.
10 (aged care adj1 (staff or worker* or nurse* or personnel)).ti,ab.
11 health personnel/ or allied health personnel/ or nursing assistants/ or physical therapist assistants/ or Physical Therapists/
12 or/8–11
13 and/3,7,12
14 limit 13 to yr="2014-Current"

Embase:

1 geriatrics/ or assisted living facility/ or home for the aged/ or nursing home/ or intermediate care facilities/ or skilled nursing facilities/ or long term care/
2 (Aged care or elderly care or residential care or residential facilit* or long-term care or nursing care facilit* or old age home* or nursing home* or assisted living facilit* or home* for the aged or housing for the elderly or residential aged care or residential aged care facilit*).ti,ab.
3 or/1–2
4 mental stress/ or burnout/ or professional burnout/ or psychological well-being/
5 ((mental or emotional or psychological) adj1 (wellbeing or health or wellness*)).ti,ab.
6 (mental health or burnout or anxiety or stress or distress or fatigue or depression).ti,ab.
7 or/4–6
8 health care personnel/ or nurse/
9 (Health personnel or health* workers* or nurses or care worker or professional carer*).ti,ab.
10 (aged care adj1 (staff or worker* or nurse* or personnel)).ti,ab.
11 health care personnel/ or paramedical personnel/ or nursing assistant/ or physiotherapist assistant/ or physiotherapist/
12 or/8–11
13 and/3,7,12
14 limit 13 to "remove medline records"
15 limit 14 to conference abstract
16 14 not 15
17 limit 16 to yr="2014-Current"

PsychINFO:

1 geriatrics/ or assisted living facilities/ or residential care institutions/ or nursing homes/ or intermediate care facilities/ or skilled nursing facilities/ or Long-Term Care/
2 (Aged care or elderly care or residential care or residential facilit* or long-term care or nursing care facilit* or old age home* or nursing home* or assisted living facilit* or home* for the aged or housing for the elderly or residential aged care or residential aged care facilit*).ti,ab.
3 or/1–2
4 psychological stress/ or burnout/ or well being.mp. [mp=title, abstract, heading word, table of contents, key concepts, original title, tests & measures, mesh word]
5 ((mental or emotional or psychological) adj1 (wellbeing or health or wellness*)).ti,ab.
6 (mental health or burnout or anxiety or stress or distress or fatigue or depression).ti,ab.
7 or/4–6
8 health personnel/ or nurses/
9 (Health personnel or health* workers* or nurses or care worker or professional carer*).ti,ab.
10 (aged care adj1 (staff or worker* or nurse* or personnel)).ti,ab.
11 health personnel/ or allied health personnel/ or nursing assistants/ or physical therapist assistants/ or Physical Therapists/
12 or/8–11
13 and/3,7,12
14 limit 13 to "remove medline records"
15 limit 14 to yr="2014-Current"

Scopus:

(TITLE-ABS-KEY (("Health personnel" OR "health* workers*" OR nurses OR "care worker*" OR "professional carer*")) OR TITLE-ABS-KEY (("aged care") PRE/1 (staff OR worker* OR nurse* OR personnel)) AND TITLE-ABS-KEY (("mental health" OR burnout OR anxiety OR stress OR distress OR fatigue OR depression)) OR TITLE-ABS-KEY ((mental OR emotional OR psychological) PRE/1 (wellbeing OR health OR wellness)) AND TITLE-ABS-KEY ("Aged care" OR "elderly care" OR "residential care" OR "residential facilit*" OR "long-term care" OR "nursing care facilit*" OR "old age home*" OR "nursing home*" OR "assisted living facilit*" OR "home* for the aged" OR "housing for the elderly" OR "residential aged care" OR "residential aged care facilit*")) AND PUBYEAR > 2013 AND PUBYEAR < 2025 AND (LIMIT-TO (LANGUAGE , "English"))

CINAHL:

1 (MH "Geriatrics") OR (MH "Assisted Living") OR (MH "Residential Facilities") OR (MH "Nursing Homes") OR (MH "Skilled Nursing Facilities") OR (MH "Long Term Care")
2 TI {[("Aged care" or "elderly care" or "residential care" or "residential facilit*" or "long-term care" or "nursing care facilit*" or "old age home*" or "nursing home*" or "assisted living facilit*" or "home* for the aged" or "housing for the elderly" or "residential aged care" or "residential aged care facilit*")]} OR AB {[("Aged care" or "elderly care" or "residential care" or "residential facilit*" or "long-term care" or "nursing care facilit*" or "old age home*" or "nursing home*" or "assisted living facilit*" or "home* for the aged" or "housing for the elderly" or "residential aged care" or "residential aged care facilit*")]}

3	(MH "Stress, Psychological") OR (MH "Burnout, Professional") OR (MH "Psychological Well-Being") OR (MH "Psychological Well-Being (Iowa NOC)+")
4	TI {[(mental or emotional or psychological) W1 (wellbeing or health or wellness)]} OR AB {[(mental or emotional or psychological) W1 (wellbeing or health or wellness)]}
5	TI [("mental health" or burnout or anxiety or stress or distress or fatigue or depression)] OR AB [("mental health" or burnout or anxiety or stress or distress or fatigue or depression)]
6	(MH "Health Personnel") OR (MH "Nursing Home Personnel") OR (MH "Gerontology Nurses") OR (MH "Long Term Care Nurses") OR (MH "Nurses") OR (MH "Physical Therapists") OR (MH "Certified Nursing Assistants") OR (MH "Physical Therapist Assistants") OR (MH "Occupational Therapy Assistants")
7	TI {[("Health personnel" or "health* workers*" or nurses or "care worker*" or "professional carer*")] OR AB [("Health personnel" or "health* workers*" or nurses or "care worker*" or "professional carer*")]}
8	TI [("aged care") W1 (staff or worker* or nurse* or personnel)] OR AB [("aged care") W1 (staff or worker* or nurse* or personnel)]
9	1 OR 2
10	3 OR 4 OR 5
11	6 OR 7 OR 8
12	9 AND 10 AND 11
13	Narrow by Language: - English
14	Limit by Publication Date: 20140101–20241231

Supplementary File 3. Coding framework.

Micro-level factors:

1) Personal Factors

• Demographics	age, gender, marital status, race
• Personal attitudes and values	ageism, self-endangering cognitions/behaviour & altruistic values, PE meaning, aversion to residents, nurse attitudes
• Physical and emotional health	self-esteem, health status, insomnia/sleep, exercise/nutrition, health-related resources
• Work-life balance	work-family culture, work Interference with family, work/family conflict
• Relationships and social support	loneliness, support from family/friends, social support – outside of work, marital status
• Personal/family stress	personal/family stress

2) Work engagement and satisfaction:

• Job satisfaction	satisfaction with working hours, job satisfaction, job dissatisfaction, satisfaction with working hours
• Organizational commitment	organizational identification, organizational commitment, work engagement
• Satisfaction with care quality	care quality perception, satisfaction with quality of care
• Satisfaction with salary	perceived fair pay, perceived financial position

3) Skills and abilities:

• Work competency / experience	years of experience, professional experience, months of employment, confidence in geriatric care, knowledge about aging, skills and knowledge, seniority
• Coping skills	adaptive coping, coping resources, experiential avoidance, resilience
• Life orientation	sense of coherence

Meso-level factors:

1) Job demands:

• Physical and emotional demands	physical demands, social pressure from work, job demands, emotional demands, psychological demands, occupational stress, mental stress, job strain
• Workload	workload stress, job-level workload, workload, work overload, work intensity
• Resident-related factors	patient behavior, agitation/aggression, verbal abuse, physical safety
• Time pressure	tight work schedule, rushed care, time pressure, enough time to care for patients, missed care
• Working hours	working hours, work shift arrangements
• Working hours	working hours, work shift arrangements
• Roles and responsibilities	work seniority, occupational position
• Work stress related to pandemics	fear of covid, covid cases in workplace, additional workload

2) Job control:

• Job autonomy / control	job autonomy, job control, decision authority, job crafting, decision latitude
• Empowerment to modify work	empowerment to modify work, PE self- determination
• Job complexity	mental regulation requirements

3) Workplace resources:

• Staffing and resources	staffing/resource adequacy, lack of staff and PPE, structural resources, staffing levels, availability of resources and/or materials
• Organizational slack	organizational slack-staff, organizational slack-space, organizational slack-time

4) Professional relations:

• Co-worker support	support from co-workers, work-related co-worker support, work-related support
• Culture and team collaboration	culture and social capital, social interactions, collegial nurse-physician relationships, social relations at work, collaboration with

team colleagues

5) Leadership:

• Management support	Relationship with superiors, support from supervisors, supervisor support, Work-related supervisor support, supervisory support, management trust
• Leadership engagement	organizational communication and participation, participation in organization affairs, management commitment
• Leadership style	Managerial domination, providing autonomy

6) Professional development:

• Job preparation	lack of job preparation, adequate job orientation
• Job training	training
• Rewards and incentives	rewards e.g., opportunities for promotion, job prospects

Macro-level factors:

1) Policy and regulation:

• National and state policies	salary, productivity standards, compensation systems
• Covid-specific factors	incoming changes during pandemic, e.g., periodic PCR tests

2) Structure and governance

• Ownership	private/public, foundation-affiliated, corporation-affiliated
• Facility size	institution size
• Location / health region	province / health region
• Service type	community care, residential aged care

Supplementary File 4. Included studies.

Authors	Year	Title	Journal	Country	Factors/ Intervention	Study design
Alenius, M. and Graf, P.	2016	Use of Electronic Medication Administration Records to Reduce Perceived Stress and Risk of Medication Errors in Nursing Homes	CIN: Computers, informatics, nursing	Sweden	Intervention	Intervention
Ali, H., Ahmed, A. and Cole, A.	2021	Capturing nurses' perception of communicative technologies in nursing facilities: Survey instrument development	International Journal of Older People Nursing	USA	Explanatory factors	Cross-sectional
Alperson, S. and Fowler, C.	2017	Holistic Self-Care: Tai Chi, Qigong Training for Caregivers at an Alzheimer Disease Assisted Living Facility	Annals of Long-Term Care	USA	Intervention	Intervention
Altintas, E., Boudoukha, A. H., Karaca, Y., Lizio, A., Luyat, M., Gallouj, K. and El Haj, M.	2022	Fear of COVID-19, emotional exhaustion, and care quality experience in nursing home staff during the COVID-19 pandemic	Archives of Gerontology and Geriatrics	Portugal	Explanatory factors	Cross-sectional
Andrade, C. and Neves, P. C.	2022	Emotional exhaustion in female health support workers in elderly care facilities	Women's Studies International Forum	Netherlands	Explanatory factors	Cross-sectional
Awosoga, O.A., Odole, A.C., Onyeso, O.K., Doan, J., Nord, C., Nwosu, I.B., Steinke, C., Ojo, J.O., Ekediegwu, E.C. and Murphy, S.	2023	Well-being of professional older adults' caregivers in Alberta's assisted living and long-term care facilities: a cross-sectional study	BMC Geriatrics	Canada	Explanatory factors	Cross-sectional
Barbosa, A., Nolan, M., Sousa, L. and Figueiredo, D.	2015	Supporting direct care workers in dementia care: Effects of a psychoeducational intervention	American Journal of Alzheimer's Disease and other Dementias	Portugal	Intervention	Intervention
Barbosa, A., Nolan, M., Sousa, L., Marques, A. and Figueiredo, D.	2016	Effects of a Psychoeducational Intervention for Direct Care Workers Caring for People With Dementia	American Journal of Alzheimer's Disease & Other Dementias	Portugal	Intervention	Intervention
Bielderman, A., Nieuwenhuis, A., Hazelhof, T. J. G. M., van Gaal, B. G. I., Schoonhoven, L., Akkermans, R. P., Spijker, A., Koopmans, R. T. C. M. and Gerritsen, Debby L.	2021	Effects on staff outcomes and process evaluation of the educating nursing staff effectively (TENSE) program for managing challenging behavior in nursing home residents with dementia: A cluster-randomized controlled trial	International Journal of Nursing Studies	Netherlands	Intervention	Intervention
Blanco-Donoso, L. M., Moreno-Jiménez, J., Amutio, A., Gallego-Alberto, L., Moreno-Jiménez, B. and Garrosa, E.	2021	Stressors, Job Resources, Fear of Contagion, and Secondary Traumatic Stress Among Nursing Home Workers in Face of the COVID-19: The Case of Spain	Journal of Applied Gerontology	Canada	Explanatory factors	Cross-sectional
Bluth, K., Lathren, C., Silbersack Hickey, J. V. T., Zimmerman, S., Wretman, C. J. and Sloane, P. D.	2021	Self-compassion training for certified nurse assistants in nursing homes	Journal of the American Geriatrics Society	USA	Intervention	Intervention
Buruck, G., Dörfel, D., Kugler, J. and Brom, S. S	2016	Enhancing well-being at work: The role of emotion regulation skills as personal resources	Journal of Occupational Health Psychology	Germany	Intervention	Intervention
Chamberlain, S.A., Gruneir, A., Hoben, M.,	2017	Influence of organizational context on nursing home	International Journal of	Canada	Explanatory	Cross-sectional

Squires, J.E., Cummings, G.G. and Estabrooks, C.A.		staff burnout: A cross-sectional survey of care aides in Western Canada	Nursing Studies		factors	
Cho, E., Lee, K.H., Kang, B., Jang, J., Shin, J., Eltaybani, S., Yamamoto-Mitani, N. and Kim, M.J.	2023	Perceived Work Environment, Educational Status, Staffing Levels, and Work Outcomes in Long-Term Care Settings During COVID-19	Journal of the American Medical Directors Association	USA	Explanatory factors	Cross-sectional
Cho, E., Min, D., Heo, S.J., Lee, K. and Kim, H.	2023	Effects of registered nurses' staffing levels, work environment and education levels on nursing home residents' quality of life and nurse outcomes	Journal of Clinical Nursing (John Wiley & Sons, Inc.)	South Korea	Explanatory factors	Cross-sectional
Ciampa, V., Steffens, Niklas K., Schuh, S.C., Fraccaroli, F. and van Dick, R.	2019	Identity and stress: an application of the expanded model of organisational identification in predicting strain at work	Work & Stress	Italy	Explanatory factors	Cross-sectional
Clausen, T., Christensen, K. B. and Nielsen, K.	2015	Does group-level commitment predict employee well-being? A prospective analysis	Journal of Occupational and Environmental Medicine	UK	Explanatory factors	Longitudinal/Cohort
Coleman, A., McLaughlin, E. and Floren, M.	2024	Practitioner Burnout and Productivity Levels in Skilled Nursing and Assisted Living Facilities, Part 1: A Descriptive Quantitative Account	American Journal of Occupational Therapy	USA	Explanatory factors	Cross-sectional
DeGraves, B.S.,Titley, H., Duan, Y., Thorne, T.E., Banerjee, S., Ginsburg, L., Salma, J., Hegadoren, K., Angel, C., Keefe, J., Lanius, R. and Estabrooks, C.A.	2024	Workforce resilience supporting staff in managing stress: A coherent breathing intervention for the long-term care workforce	Journal of the American Geriatrics Society	Canada	Intervention	Intervention
Dhaini SR, Zúñiga F, Ausserhofer D, Simon M, Kunz R, De Geest S, and Schwendimann R.	2016	Care workers health in Swiss nursing homes and its association with psychosocial work environment: A cross-sectional study	International Journal of Nursing Studies	Switzerland	Explanatory factors	Cross-sectional
Dichter, M. N., Trutschel, D., Schwab, C. G. G., Haastert, B., Quasdorf, T. and Halek, M.	2017	Dementia care mapping in nursing homes: Effects on caregiver attitudes, job satisfaction, and burnout. A quasi-experimental trial	International Psychogeriatrics	Germany	Intervention	Intervention
Dreher, M.M., Hughes, R.G., Handley, P.A. and Tavakoli, A.S.	2019	Improving Retention Among Certified Nursing Assistants Through Compassion Fatigue Awareness and Self-Care Skills Education	Journal of Holistic Nursing	USA	Intervention	Intervention
Duan, Y., Song, Y., Thorne, T.E., Iaconi, A., Norton, P.G. and Estabrooks, C.A.	2023	The Complexity of Burnout Experiences among Care Aides: A Person-Oriented Approach to Burnout Patterns	Healthcare (2227-9032)	Canada	Explanatory factors	Cross-sectional
Eder, L.L. and Meyer, B.	2023	The role of self-endangering cognitions between long-term care nurses' altruistic job motives and exhaustion	Frontiers in Health Services	Germany	Explanatory factors	Cross-sectional and longitudinal
Edvardsson, D., Sandman, P. O. and Borell, L.	2014	Implementing national guidelines for person-centered care of people with dementia in residential aged care: effects on perceived person-centeredness, staff strain, and stress of conscience	International Psychogeriatrics	Sweden	Intervention	Intervention
Elliott, K.-E. J., Rodwell, J. and Martin, A.J.	2017	Aged care nurses' job control influence satisfaction and mental health	Journal of Nursing Management	Australia	Explanatory factors	Cross-sectional
Elovainio, M., Heponiemi, T., Kuusio, H., Jokela, M., Aalto, A.-M., Pekkarinen, L., and	2015	Job demands and job strain as risk factors for employee wellbeing in elderly care: an instrumental-	European journal of public health	Finland	Explanatory factors	Cross-sectional

Noro, A., Finne-Soveri, H., Kivimaki, M. and Sinervo, T.		variables analysis				
Ericson-Lidman, E. and Åhlin, J.	2017	Assessments of Stress of Conscience, Perceptions of Conscience, Burnout, and Social Support Before and After Implementation of a Participatory Action-Research-Based Intervention	Clinical Nursing Research	Sweden	Intervention	Intervention
Ertan, ŞS and Şeşen, H.	2022	Positive organizational scholarship in healthcare: The impact of employee training on performance, turnover, and stress	Journal of Management and Organization	Cyprus	Explanatory factors	Cross-sectional
Faretta, E., Garau, M.I., Gallina, E., Pagani, M. and Fernandez, I.	2022	Supporting healthcare workers in times of COVID-19 with eye movement desensitization and reprocessing online: A pilot study	Frontiers in psychology	Italy	Intervention	Intervention
Fengsong, G., Newcombe, P., Tilse, C., Wilson, J. and Tuckett, A.	2014	Models for predicting turnover of residential aged care nurses: A structural equation modelling analysis of secondary data	International Journal of Nursing Studies	Australia	Explanatory factors	Cross-sectional
Fukuda, K., Hattori, H., Toba, K., Terada, S., Hashimoto, M., Ukai, K., Kumagai, R., Suzuki, M., Nagaya, M., Yoshida, M. and Murotani, K.	2018	Effectiveness of educational program using printed educational material on care burden distress among staff of residential aged care facilities without medical specialists and/or registered nurses: Cluster quasi-randomization study	Geriatrics & Gerontology International	Japan	Intervention	Intervention
Fushimi, M.	2019	Depressive symptoms and related factors among workers in care institutions for older persons in Japan: a cross-sectional study	European Geriatric Medicine	Japan	Explanatory factors	Cross-sectional
Gao, F., Newcombe, P., Tilse, C., Wilson, J. and Tuckett, A.	2017	Challenge-related stress and felt challenge: Predictors of turnover and psychological health in aged care nurses	Collegian	Australia	Explanatory factors	Longitudinal/Cohort
Gillis, K., van Diermen, L., Lips, D., Lahaye, H., De Witte, M., Van Wiele, L., Roelant, E., Hockley, J. and Van Bogaert, P.	2024	The impact of need-based care on formal caregivers' wellbeing in nursing homes: A cluster randomized controlled trial	International Journal of Nursing Studies	Belgium	Intervention	Intervention
Halek, M., Reuther, S., Müller-Widmer, R., Trutschel, D. and Holle, D.	2020	Dealing with the behaviour of residents with dementia that challenges: A stepped-wedge cluster randomized trial of two types of dementia-specific case conferences in nursing homes (FallDem)	International Journal of Nursing Studies	Germany	Intervention	Intervention
Halperin, D.	2020	The association between work stressors, knowledge about aging, burnout, and job satisfaction among nursing home activity directors	Activities, Adaptation & Aging	Israel	Explanatory factors	Cross-sectional
Hering, C., Gangnus, A., Budnick, A., Kohl, R., Steinhagen-Thiessen, E., Kuhlmey, A. and Gellert, P.	2022	Psychosocial burden and associated factors among nurses in care homes during the COVID-19 pandemic: findings from a retrospective survey in Germany	BMC Nursing	Germany	Explanatory factors	Cross-sectional
Honda, A., Fauth, E.B., Liu, Y. and Honda, S.	2022	Predictors of EffortÃ¢â,¬â€œReward Imbalance Among Employees Providing Three Types of Long-Term Care Services in Japan: Implications for Employee Well-Being	Journal of Applied Gerontology	Japan	Explanatory factors	Cross-sectional

Hyunju, L., Youngja, S., Jihye, K., Hye Young, S., Jinhee, P. and Youngran, Y.	2023	The impact of Long COVID, work stress related to infectious diseases, fatigue, and coping on burnout among care providers in nursing home: A cross-sectional correlation study	Journal of Korean Gerontological Nursing	South Korea	Explanatory factors	Cross-sectional
Iecovich, E., and Avivi, M.	2017	Agism and burnout among nurses in long-term care facilities in Israel	Aging & Mental Health	Israel	Explanatory factors	Cross-sectional
Ikeda, S., Eguchi, H., Hiro, H., Mafune, K., Koga, K., Nishimura, K. and Nakashima, M.	2021	Work-Family Spillover, Job Demand, Job Control, and Workplace Social Support Affect the Mental Health of Home-Visit Nursing Staff	Journal of UOEH	Japan	Explanatory factors	Cross-sectional
Islam, M.S., Baker, C., Huxley, P., Russell, I.T. and Dennis, M.S.	2017	The nature, characteristics and associations of care home staff stress and wellbeing: a national survey	BMC Nursing	UK	Explanatory factors	Cross-sectional
Jakobsen, L. M., Jorgensen, A. F. B., Thomsen, B. L., Albertsen, K., Greiner, B. A. and Rugulies, R.	2016	Emotion work within eldercare and depressive symptoms: A cross-sectional multi-level study assessing the association between externally observed emotion work and self-reported depressive symptoms among Danish eldercare workers	International Journal of Nursing Studies	Denmark	Explanatory factors	Cross-sectional
Jakobsen, L. M., Jorgensen, A. F. B., Thomsen, B. L., Greiner, B. A. and Rugulies, R.	2015	A multilevel study on the association of observer-assessed working conditions with depressive symptoms among female eldercare workers from 56 work units in 10 care homes in Denmark	BMJ open	Denmark	Explanatory factors	Cross-sectional
Jameson, S. and Parkinson, L.	2022	Work-related well-being of personal care attendants employed in the aged care sector: Prevalence and predictors of compassion fatigue	Australasian Journal on Ageing	Australia	Explanatory factors	Cross-sectional
Jenull, B. B. and Wiedermann, W.	2015	The Different Facets of Work Stress: A Latent Profile Analysis of Nurses' Work Demands	Journal of Applied Gerontology	Austria	Explanatory factors	Cross-sectional
Jones, K. F., Kearney, M. and Best, M. C.	2024	Effect of a Spiritual Care Training Program to Build Knowledge, Competence, Confidence and Self-awareness Among Australian Health and Aged Care Staff: An Exploratory Study	Journal of Religion & Health	Australia	Intervention	Intervention
Jun-Ah, S. and Younjae, O.	2015	The Association Between the Burden on Formal Caregivers and Behavioral and Psychological Symptoms of Dementia (BPSD) in Korean Elderly in Nursing Homes	Archives of Psychiatric Nursing	South Korea	Explanatory factors	Cross-sectional
Kada, O.	2019	Hospital Transfers of Nursing Home Residents: A Vignette Experiment on Nurses' Decision Making	Journal of Applied Gerontology	Austria	Explanatory factors	Cross-sectional
Kandelman, N., Mazars, T. and Levy, A.	2018	Risk factors for burnout among caregivers working in nursing homes	Journal of Clinical Nursing (John Wiley & Sons, Inc.)	France	Explanatory factors	Cross-sectional
Kim, B. J. and Lee, S. Y.	2021	A cross-sectional study on the impacts of perceived job value, job maintenance, and social support on burnout among long-term care staff in hawaii	International Journal of Environmental Research and Public Health	USA	Explanatory factors	Cross-sectional
Kim, B. J., Ishikawa, H., Liu, L., Ohwa, M., Sawada, Y., Lim, H. Y., Kim, H. Y., Choi, Y.	2018	The effects of job autonomy and job satisfaction on burnout among careworkers in long-term care settings:	Educational Gerontology	South Korea	Explanatory factors	Cross-sectional

and Cheung, C.		Policy and practice implications for Japan and South Korea				
Kim, B., Liu, L., Ishikawa, H. and Park, S.-H.	2019	Relationships between social support, job autonomy, job satisfaction, and burnout among care workers in long-term care facilities in Hawaii	Educational Gerontology	USA	Explanatory factors	Cross-sectional
Kim, B.J. and Choi, C.J.W.	2023	Impact of compensation and willingness to keep same career path on burnout among long-term care workers in Japan	Human Resources for Health	Japan	Explanatory factors	Cross-sectional
Kloos, N., Drossaert, C. H. C., Bohlmeijer, E. T. and Westerhof, G. J.	2019	Online positive psychology intervention for nursing home staff: A cluster-randomized controlled feasibility trial of effectiveness and acceptability	International Journal of Nursing Studies	Netherlands	Intervention	Intervention
Korbus, H., Hildebrand, C., Schott, N., Bischoff, L., Otto, A.-K., Joellenbeck, T., Schoene, D., Voelcker-Rehage, C., Vogt, L., Weigelt, M. and Wollesen, B.	2023	Health status, resources, and job demands in geriatric nursing staff: A cross-sectional study on determinants and relationships	International Journal of Nursing Studies	Germany	Explanatory factors	Cross-sectional
Kossek, E. E., Thompson, R. J., Lawson, K. M., Bodner, T., Perrigino, M. B., Hammer, L. B., Buxton, O. M., Almeida, D. M., Moen, P., Hurtado, D. A., Wipfli, B., Berkman, L. L., Bray, J. W. and Berkman, L. F.	2019	Caring for the elderly at work and home: Can a randomized organizational intervention improve psychological health?	Journal of Occupational Health Psychology	USA	Intervention	Intervention
Kubicek, B. and Korunka, C.	2015	Does job complexity mitigate the negative effect of emotion-rule dissonance on employee burnout?	Work & Stress	Austria	Explanatory factors	Longitudinal/Cohort
Kubicek, B., Korunka, C. and Tement, S.	2014	Too much job control? Two studies on curvilinear relations between job control and eldercare workers' well-being	International Journal of Nursing Studies	Slovenia	Explanatory factors	Cross-sectional and longitudinal
Kukihara, H., Ando, M. and Yamawaki, N.	2022	The effects of yoga and mindful meditation on elderly care worker's burnout: a CONSORT-compliant randomized controlled trial	Journal of rural medicine (JRM)	Japan	Intervention	Intervention
Lee, S. and Oh, G.	2023	Working Conditions Affecting Home Care Workers' Stress and Turnover Intention	Journal of Applied Gerontology	South Korea	Explanatory factors	Cross-sectional
Levy, A. M.,Grigorovich, A., McMurray, J., Quirt, H., Ranft, K., Engell, K., Stewart, S., Astell, A., Kokorelias, K., Schon, D., Rogrigues, K., Tsokas, M., Flint, A. J. and Iaboni, A.	2024	Implementation of the Dementia Isolation Toolkit in long-term care improves awareness but does not reduce moral distress amongst healthcare providers	BMC health services research	Canada	Intervention	Intervention
Lin, W.-Y., Chen, Y.-A., Huang, K.-H., Tsai, T.-H. and Shieh, S.-H.	2024	Depression and anxiety between nurses and nursing assistants working in long-term care facilities during the COVID-19 pandemic	International nursing review	Taiwan	Explanatory factors	Cross-sectional
Low, Y.S., Bhar, S. and Chen, W.S.	2022	Exploring the relationship between co-worker and supervisor support, self- confidence, coping skills and burnout in residential aged care staff	BMC Nursing	Australia	Explanatory factors	Cross-sectional
Muntaner, C., Ng, E., Prins, S. J., Bones-Rocha, K., Espelt, A. and Chung, H.	2015	Social Class and Mental Health: Testing Exploitation as a Relational Determinant of Depression	International Journal of Health Services	USA	Explanatory factors	Cross-sectional

Navarro Prados, A.B., Jiménez García-Tizón, S. and Meléndez, J.C.	2022	Sense of coherence and burnout in nursing home workers during the COVID-19 pandemic in Spain	Health & Social Care in the Community	Spain	Explanatory factors	Cross-sectional
Navarro-Prados, A.B., García-Tizón, S.J., Meléndez, J.C. and López, J.	2024	Factors associated with satisfaction and depressed mood among nursing home workers during the covidÃ¢â,¬Â19 pandemic	Journal of Clinical Nursing (John Wiley & Sons, Inc.)	Spain	Explanatory factors	Cross-sectional
Navarro-Prados, A.B., Rodríguez-Ramírez, Y., Satorres, E. and Meléndez, J.C.	2024	Stress and burnout in nursing home and égida workers during COVID-19	Journal of Advanced Nursing (John Wiley & Sons, Inc.)	Spain	Explanatory factors	Cross-sectional
O'Brien, W. H., Singh, R., Horan, K., Moeller, M. T., Wasson, R. and Jex, S. M.	2019	Group-Based Acceptance and Commitment Therapy for Nurses and Nurse Aides Working in Long-Term Care Residential Settings	Journal of Alternative & Complementary Medicine	USA	Intervention	Intervention
Pérez, V., Menéndez-Crispín, E.J., Sarabia-Cobo, C., de Lorena, P., Fernández-Rodríguez, A. and González-Vaca, J.	2022	Mindfulness-Based Intervention for the Reduction of Compassion Fatigue and Burnout in Nurse Caregivers of Institutionalized Older Persons with Dementia: A Randomized Controlled Trial	International Journal of Environmental Research and Public Health	Spain	Intervention	Intervention
Rouxel, G., Michinov, E. and Dodeler, V.	2016	The influence of work characteristics, emotional display rules and affectivity on burnout and job satisfaction: A survey among geriatric care workers	International journal of nursing studies	France	Explanatory factors	Cross-sectional
Schmidt, S.G., Dichter, M.N., Bartholomeyczik, S., and Hasselhorn, H.M.	2014	The satisfaction with the quality of dementia care and the health, burnout and work ability of nurses: A longitudinal analysis of 50 German nursing homes	Geriatric Nursing	Germany	Explanatory factors	Longitudinal/Cohort
Schoultz, M., McGrogan, C., Beattie, M., Macaden, L., Carolan, C. and Dickens, G.L.	2022	Uptake and effects of psychological first aid training for healthcare workers' wellbeing in nursing homes: A UK national survey	PLoS ONE	UK	Intervention	Intervention
Squires, J.E., Baumbusch, J., Demery Varin, M., MacDonald, I., Chamberlain, S., Bostrom, A.-M., Thompson, G., Cummings, G. and Estabrooks, C.A.	2019	A Profile of Regulated Nurses Employed in Canadian Long-Term Care Facilities	Canadian journal on aging = La revue canadienne du vieillissement	Canada	Explanatory factors	Longitudinal/Cohort
Tanaka, K., Iso, N., Sagari, A., Tokunaga, A., Iwanaga, R., Honda, S., Nakane, H., Ohta, Y. and Tanaka, G.	2015	Burnout of Long-term Care Facility Employees: Relationship with Employees' Expressed Emotion Toward Patients	International Journal of Gerontology	Japan	Explanatory factors	Cross-sectional
Vogel, B., De Geest, S., Fierz, K., Beckmann, S. and Zúñiga, F.	2016	Dementia care worker stress associations with unit type, resident, and work environment characteristics: a cross-sectional secondary data analysis of the Swiss Nursing Homes Human Resources Project (SHURP)	International Psychogeriatrics	Switzerland	Explanatory factors	Cross-sectional
White, E.M., Aiken, L.H., Sloane, D.M. and McHugh, M.D.	2020	Nursing home work environment, care quality, registered nurse burnout and job dissatisfaction	Geriatric Nursing	USA	Explanatory factors	Cross-sectional
Wollesen, B., Hagemann, D., Pabst, K., Schlüter, R., Bischoff, L. L., Otto, A. K., Hold, C. and Fenger, A.	2019	Identifying individual stressors in geriatric nursing staff-a cross-sectional study	International Journal of Environmental Research and Public Health	Germany	Explanatory factors	Cross-sectional
Woodhead, E.L. and Northrop, L. and Edelstein, B.	2016	Stress, Social Support, and Burnout among Long-Term Care Nursing Staff	Journal of Applied Gerontology	USA	Explanatory factors	Cross-sectional
Wu, Q., Yamaguchi, Y. and Greiner, C.	2022	Factors Related to Mental Health of Foreign Care	International Journal of	Japan	Explanatory	Cross-sectional

		Workers in Long-Term Care Facilities in Japan during the COVID-19 Pandemic-A Comparative Study	Environmental Research and Public Health		factors	
Yeatts, D. E., Seckin, G., Thompson, M., Auden, D., Cready, C. and Shen, Y.	2018	Burnout among direct-care workers in nursing homes: Influences of organisational, workplace, interpersonal and personal characteristics	Journal of Clinical Nursing (John Wiley & Sons, Inc.)	USA	Explanatory factors	Cross-sectional
Yepes-Baldo, M., Romeo, M., Westerberg, K. and Nordin, M.	2018	Job Crafting, Employee Well-being, and Quality of Care	Western Journal of Nursing Research	Spain	Explanatory factors	Cross-sectional
Yia-Wun, L., Yenping, H., Yu-Hsiu, L., Wen-Yi, C.	2014	The impact of job Stressors on health-related quality of life of nursing assistants in long-term care settings	Geriatric Nursing	Taiwan	Explanatory factors	Cross-sectional
Young, C.C., Kesler, S., Walker, V.G., Johnson, A. and Harrison, T.C.	2023	An Online Mindfulness-based Intervention for Certified Nursing Assistants in Long-term Care	Journal of Holistic Nursing	USA	Intervention	Intervention
Zhang, Y., Punnett, L. and Nannini, A.	2017	Work-Family Conflict, Sleep, and Mental Health of Nursing Assistants Working in Nursing Homes	Workplace Health & Safety	USA	Explanatory factors	Cross-sectional
Zhang, Y., Punnett, L., Mawn, B. and Gore, R.	2016	Working Conditions and Mental Health of Nursing Staff in Nursing Homes	Issues in Mental Health Nursing	USA	Explanatory factors	Cross-sectional
Zhao, X., Song, Y., Shi, X., Su, Y., Zhang, W., Si, H. and Zhu, L.	2022	Relationship between depressive symptoms and subjective occupational well-being among nursing home staff: the mediating role of resilience	Archives of Psychiatric Nursing	China	Explanatory factors	Cross-sectional
Zwakhalen, S. M. G., Hamers, J. P. H., van Rossum, E., Ambergen, T., Kempen, G. I. J. M. and Verbeek, H.	2018	Working in small-scale, homelike dementia care: effects on staff burnout symptoms and job characteristics. A quasi-experimental, longitudinal study	Journal of Research in Nursing	Netherlands	Explanatory factors	Longitudinal/Cohort

AIMS Press

© 2025 the Author(s), licensee AIMS Press. This is an open access article distributed under the terms of the Creative Commons Attribution License (https://creativecommons.org/licenses/by/4.0)
